# Challenging Scenarios and Debated Indications for Laparoscopic Liver Resections for Hepatocellular Carcinoma

**DOI:** 10.3390/cancers15051493

**Published:** 2023-02-27

**Authors:** Giammauro Berardi, Edoardo Maria Muttillo, Marco Colasanti, Germano Mariano, Roberto Luca Meniconi, Stefano Ferretti, Nicola Guglielmo, Marco Angrisani, Alessio Lucarini, Eleonora Garofalo, Davide Chiappori, Ludovica Di Cesare, Damiano Vallati, Paolo Mercantini, Giuseppe Maria Ettorre

**Affiliations:** 1 Department of General, Hepatobiliary and Pancreatic Surgery, Liver Transplantation Service, San Camillo Forlanini Hospital of Rome, 00152 Rome, Italy; 2 Surgical and Medical Department of Translational Medicine, Sant’Andrea Hospital, Sapienza University of Rome, 00189 Rome, Italy

**Keywords:** laparoscopic liver resection, hepatocellular carcinoma, advanced cirrhosis, portal hypertension, large HCC, posterosuperior segments, recurrent HCC

## Abstract

**Simple Summary:**

Minimally invasive liver resections are nowadays performed worldwide for both benign and malignant lesions. Good short-term and safe long-term outcomes have been reported. Despite this growing implementation of the technique, challenging scenarios and debated indications still exist. There is currently a lack of high-quality evidence regarding minimally invasive liver resections in portal hypertension, advanced cirrhosis, lesions in the posterosuperior segments and large and recurrent tumors.

**Abstract:**

Laparoscopic liver resections (LLRs) have been increasingly adopted for the treatment of hepatocellular carcinoma (HCC), with safe short- and long-term outcomes reported worldwide. Despite this, lesions in the posterosuperior segments, large and recurrent tumors, portal hypertension, and advanced cirrhosis currently represent challenging scenarios in which the safety and efficacy of the laparoscopic approach are still controversial. In this systematic review, we pooled the available evidence on the short-term outcomes of LLRs for HCC in challenging clinical scenarios. All randomized and non-randomized studies reporting LLRs for HCC in the above-mentioned settings were included. The literature search was run in the Scopus, WoS, and Pubmed databases. Case reports, reviews, meta-analyses, studies including fewer than 10 patients, non-English language studies, and studies analyzing histology other than HCC were excluded. From 566 articles, 36 studies dated between 2006 and 2022 fulfilled the selection criteria and were included in the analysis. A total of 1859 patients were included, of whom 156 had advanced cirrhosis, 194 had portal hypertension, 436 had large HCCs, 477 had lesions located in the posterosuperior segments, and 596 had recurrent HCCs. Overall, the conversion rate ranged between 4.6% and 15.5%. Mortality and morbidity ranged between 0.0% and 5.1%, and 18.6% and 34.6%, respectively. Full results according to subgroups are described in the study. Advanced cirrhosis and portal hypertension, large and recurrent tumors, and lesions located in the posterosuperior segments are challenging clinical scenarios that should be carefully approached by laparoscopy. Safe short-term outcomes can be achieved provided experienced surgeons and high-volume centers.

## 1. Introduction

Hepatocellular carcinoma (HCC) is the most common primary liver tumor and the third leading cause of cancer-related deaths worldwide [1,2]. Whenever feasible, liver resection (LR) is one of the treatments of choice in very early and early-stage disease, offering long-term survivals exceeding 50% at 5 years [3]. Since 1992, when the first laparoscopic liver resection (LLR) was described, minimally invasive approaches have been increasingly employed for both benign and malignant liver diseases [4]. Indeed, despite the initial skepticism from the oncological point of view, nowadays, LLRs are considered safe for the treatment of malignant tumors and are widely adopted in experienced centers for colorectal liver metastases, intrahepatic cholangiocarcinomas, and HCCs [5]. Recent meta-analyses disclosed improved short- and comparable long-term outcomes of LLRs compared to open in the setting of HCC [6,7]. However, a variety of different patients and tumor presentations were included, eventually analyzing a heterogeneous population with different risk factors from both the perioperative and long-term standpoint.

Conditions such as advanced cirrhosis (AC), portal hypertension (PH), lesions located in the posterosuperior (PS) segments, large tumors, and recurrent HCCs represent unique clinical scenarios that require careful and specific considerations in the setting of minimally invasive approaches. Indeed, these conditions are associated with increased perioperative morbidity and mortality and were initially considered as contraindications to LLRs as recommended in the Southampton Consensus Guidelines for Laparoscopic liver surgery [8]. Despite this, experienced centers have been pushing the indications in these challenging scenarios, reporting safe outcomes both in the perioperative setting and long-term survivals [9,10,11]. Nevertheless, the evidence is still limited to small studies, which have been mostly singe center and retrospective in nature. This systematic review aimed to pool all the available literature regarding LLRs in these challenging scenarios and to summarize the evidence.

## 2. Material and Methods

### 2.1. Literature Search

Preferred Reporting Items for Systematic Reviews and Meta-Analyses (PRISMA) statement guidelines were followed for conducting and reporting this systematic review. A systematic literature search was performed independently by two of the authors (E.M.M. and G.B.) using PubMed, WoS, and Scopus databases. The search was limited to studies in humans and published in English. Case reports, reviews, and meta-analyses were excluded. No restrictions were set for the date of publication. The search strategy was based on different combinations of words for each database. For the PubMed database, the following combination was used: (repeat hepatectomy OR recurrent HCC) AND (large HCC OR large hepatocellular carcinoma) AND (laparoscopic liver resections OR minimally invasive) AND (portal hypertension) AND (advanced liver cirrhosis) AND (posterosuperior segments). The same keywords were inserted in the search manager fields of Scopus. Extensive crosschecking of the reference lists of all retrieved articles that fulfilled the inclusion criteria further broadened the search. This systematic review was registered in the PROSPERO database with the number CRD42023396942.

### 2.2. Study Selection

The same two authors independently screened the titles and abstracts of the studies that were identified with the electronic search. Duplicate studies were excluded. The following criteria were set: (1) studies reporting laparoscopic liver resections for the above-mentioned indications; (2) studies reporting at least one perioperative outcome. The following exclusion criteria were set: (1) studies reporting non-laparoscopic liver resections, (2) studies not reporting separate outcomes for laparoscopic liver resections and (3) studies in which it was impossible to retrieve or calculate the data of interest. In the case of more than one report from the same center, only the most recent or the highest-quality study was included in the review. Advanced cirrhosis was defined as a Child–Pugh score of B or more [12]. Portal hypertension was defined as the presence of indirect signs of clinically significant portosystemic shunts (radiological or biochemical) or by a portosystemic gradient of more than 10 mmHg [13]. Segments VII, VIII, and IVa were considered posterosuperior [14]. A size of >5 cm was considered a large HCC [15].

### 2.3. Data Extraction

The same two authors extracted the main data as follows: (1) first author, study type, and subgroup; (2) number and characteristics of patients including Child–Pugh and/or MELD score; (3) intraoperative characteristics including the number of major/minor hepatectomies, anatomic or non-anatomic resections, operative time, blood loss, Pringle maneuver, conversion rates, and (4) postoperative outcomes including complications, Clavien–Dindo et al. [16] grade, liver-specific complications (bile leak, ascites, and liver failure) and mortality. Liver failure was defined according to the classification of International Study Group of Liver Surgery (ISGLS) [17] Major complications were defined as Clavien–Dindo > II. Relevant texts, tables, and figures were reviewed for data extraction, and whenever further information was required, the corresponding authors of the papers were contacted by e-mail. Discrepancies between the two reviewers were resolved by consensus discussion. Quality assessment was performed according to the Newcastle–Ottawa Scale (Table 1) [18].

## 3. Results

**Figure 1:**
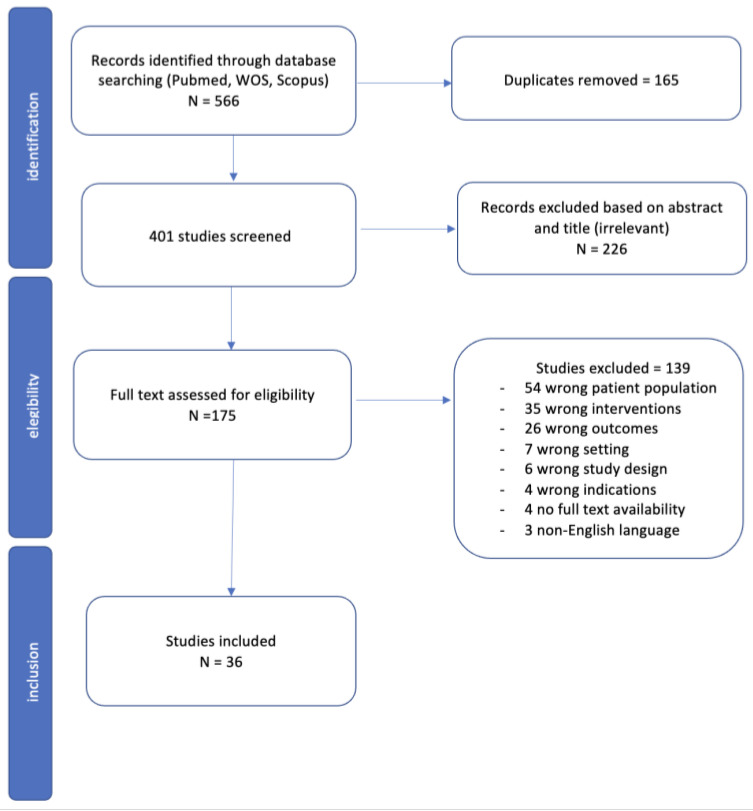
Prisma flow diagram.

The literature search yielded 566 articles; after duplicate removal, 401 titles and abstracts were reviewed (Figure 1). Of these, 226 papers were excluded based on abstract and title; 175 articles were assessed for eligibility and full text screened. Of these, 139 articles were excluded. Finally, a total of 36 articles dated between 2006 and 2022 fulfilled the selection criteria and were included in this systematic review [19,20,21,22,23,24,25,26,27,28,29,30,31,32,33,34,35,36,37,38,39,40,41,42,43,44,45,46,47,48,49,50,51,52,53,54]. There was no disagreement between the authors regarding eligibility. The articles consisted of 33 retrospective and three prospective reports, gathering a total of 1859 patients. Characteristics of the included studies are summarized in Table 2.

### 3.1. Advanced Cirrhosis

Four studies were included in the subgroup of LLRs in patients with advanced cirrhosis gathering a total of 156 patients, of whom 116 (74.4%) were male and 40 (25.6%) were female (Table 2). Median age ranged between 60 (27–79) and 68 (27–84). One-hundred and fifty patients (96.1%) were scored as Child–Pugh B and 6 (3.9%) as Child–Pugh C with a MELD score of 9 (4–22) that was reported only in one study [20]. Three studies reported the number of minor/major hepatectomies and anatomic/non-anatomic resections (Table 3). Minor hepatectomies were more frequently performed (117/131, 89.3%) as compared to major hepatectomies (14/131, 10.7%). Non-anatomic resections were performed in 74/131 (56.5%) cases, while anatomic hepatectomies were carried out in 57/131 (43.5%). Only one study described tumor localization (62% anterolateral and 38% posterosuperior segments) [20]. Operative time ranged between 99 (43–354) and 235 (84–605) minutes, while blood loss was between 50 (10–4750) and 800 (240–1000) mL (Table 3). Concerning hilar clamping, no Pringle maneuver was used in two studies, while 63/156 (40.4%) of the hepatectomies were performed under clamping among the remaining studies. Overall, 8/151 (5.3%) patients required intraoperative blood transfusions. Thirteen cases (8.3%) were converted to open. Concerning postoperative outcomes, 54 (34.6%) patients developed postoperative complications, of which 44 (81.5%) were minor and 10 (18.5%) were major (Table 4). Liver-specific morbidity was observed in 34 (21.8%) cases, with 3 (1.9%) patients experiencing liver failure, 29 (18.6%) patients experiencing ascites, and 2 (1.3%) patients experiencing bile leaks. Median hospital stay ranged between 2 (1–19) and 10 (7–15) days. Eight (5.1%) patients died within 90 days of surgery (Table 4).

#### Comparative Results between Open vs. Minimally Invasive Surgery in Advanced Cirrhosis

Only Troisi et al. compared open vs. laparoscopic surgery in advanced cirrhosis. All patients were scored as Child–Pugh B. Laparoscopy was associated with lower blood loss (median 110 mL versus 400 mL in the open group; *p* = 0.004), lower morbidity (38% vs. 51%; *p* = 0.041) and fewer major complications (7% vs. 21%; *p* = 0.010) [20].

### 3.2. Portal Hypertension

Six studies were included in the subgroup of LLRs in patients with portal hypertension with a total of 194 patients, 139 (71.6%) male and 55 (28.4%) female with a median age between 50 (29–70) and 64 (52–83). One-hundred and sixty-two (83.5%) patients were scored as Child–Pugh A, 29 (14.9%) were scored as Child–Pugh B, and 3 (1.5%) were scored as Child–Pugh C, with a MELD score of 8 (6–11) that was reported only in one study [23] (Table 2). Tumor size ranged between 2.0 (1.1–5.7) and 3.3 (2.0–4.8) cm. The majority of patients underwent a minor hepatectomy (165, 85.1%), while major hepatectomies were performed in 29 (14.9%) cases. Non-anatomic resections were conducted in 64/105 (61.0%) patients, while 41/105 (39.0%) underwent an anatomical hepatectomy (Table 3). Only one study reported tumor’s location (77% anterolateral and 23% posterosuperior segments) [27]. Operative time ranged between 150 (90–215) and 336 ± 18 min. Blood loss ranged between 90 (80–1000) and 415 (200–731) mL. Pringle maneuver was performed in 43/71 cases (60.6%). Intraoperative blood transfusions were needed in 7/89 patients (7.9%). Conversion to open happened in 8/89 (9.0%) cases (Table 3). Regarding postoperative outcomes, 69 (35.5%) patients developed postoperative complications of which 45 (71.4%) were minor and 18 (28.6%) were major. Liver-specific morbidity was reported in 16/89 (17.9%) cases with 89 (6.7%) patients developing liver failure and 10/89 (11.2%) experiencing ascites. Hospital stays ranged between 3 (2–20) and 13.5 (9–24) days. In this subgroup, neither bile leak nor 90-day mortality was observed (Table 4).

#### Comparative Results between Open vs. Minimally Invasive Surgery in Portal Hypertension

Only Ruzzenente et al. reported comparative results between laparoscopic and open surgery in portal hypertension. They found that patients undergoing laparoscopic approach had shorter hospital stay (>7 days: open 55% vs. laparoscopic 29%, *p* < 0.001) as well as lower morbidity (open: 42% vs. laparoscopic: 29%, *p* = 0.001) [28].

### 3.3. Large HCC

Seven studies were included in the subgroup of patients with large HCC, with a total of 436 individuals, 345 (79.1%) males, and 91 (20.9%) females. Age ranged between 51 ± 11.9 and 71 (61–77). Two hundred and forty-one patients (80.3%) were scored as Child–Pugh A, and 59/300 (19.7%) were scored as Child–Pugh B (Table 2). No Child–Pugh C patients were reported in this subgroup. Tumor size ranged between 6 (5.5–10) and 7.8 ± 2.15 cm. Only one study described tumor locations (73 (71.56%) anterolateral and 29 (28.44%) posterosuperior segments) [32]. Three hundred and thirty-three patients (76.4%) underwent a minor hepatectomy, while 103 (23.6%) were submitted to a major resection. Anatomic hepatectomies were performed in 277 patients (63.5%) (Table 3). Operative time ranged between 195 (90–390) and 358 ± 136 min. Pringle maneuver was applied in 10/58 (17.2%) cases. Blood loss ranged between 50 (10–1200) and 623 ± 841.7 mL. Forty-nine (13.6%) patients required intraoperative blood transfusions. Thirty five (8.0%) cases were converted to laparotomy (Table 3). Only one study described the reason for conversion (four cases for uncontrollable bleeding, two cases for oncological safety and three cases for tumor encroaching on the diaphragmatic muscle) [35]. Regarding postoperative outcomes, 87 (19.9%) patients developed complications, of which 65 (74.7%) were minor and 22 (25.3%) were major (Table 4). Liver-specific morbidity was observed in 20/398 (5.0%) cases, with 7/436 patients (1.6%) developing liver failure, 8/398 (2.0%) developing ascites, and 5/398 (1.3%) developing bile leak. Median hospital stay ranged between 6 (4–8) and 11.4 ± 3.1 days. One patient (0.2%) died within 90 days of surgery.

#### Comparative Results between Open vs. Minimally Invasive Surgery in Large HCC

Four studies compared the postoperative results of open vs. laparoscopic surgery [30,31,33,35] in the setting of large lesions. All of them showed shorter hospital stay in the laparoscopic group. Xiang et al. and Ai et al. showed lower rates of postoperative complications in the laparoscopic group [33,35]. Chiang et al. and Fu et al. found a lower blood loss [30,31]. No differences were found in terms of postoperative mortality. 

### 3.4. Posterosuperior Segments

Nine studies were included in the subgroup of LLRs in patients with HCC located in the posterosuperior segments with a total of 477 patients, 360 (75.5%) male and 117 (24.5%) female with an age ranging between 51.6 ± 10.2 and 71 (59.5–75) (Table 2). Three hundred and seventy-two patients (96.9%) were scored as Child–Pugh A, 10/384 (2.6%) were scored as Child–Pugh B and 2/384 (0.5%) were scored as Child–Pugh C. Tumor size ranged between 2.31 ± 0.78 and 4.22 ± 2.05 cm. Major hepatectomies were performed in 75 (15.7%) cases, while 402 (84.3%) underwent a minor resection. In 216 (45.2%) cases, a non-anatomic resection was performed as compared to 261 (54.8%) in which the resection was anatomic (Table 3). Operative time ranged between 215 ± 70 and 362 ± 180.7 min. Pringle maneuver was applied in 61/171 (35.7%) cases. Blood loss ranged between 55 (20–1400) and 1376 ± 2509 mL. Sixty-three (15.0%) patients required an intraoperative blood transfusion, and conversion to open was necessary in 15.5% of cases (Table 3). Regarding postoperative outcomes, 77/415 (18.6%) patients had complications of which 45 (66.2%) were minor and 23 (33.8%) were major. Liver-specific morbidity was observed in 7/161 (4.3%) cases, with 1/161 (0.6%) patient developing liver failure, 4/161 (2.4%) experiencing ascites, and 2 (1.2%) experiencing bile leaks (Table 4). Hospital stay ranged between 5 (3–7) and 10.5 ± 2.7 days. Mortality at 90 days was 0%.

#### Comparative Results between Open vs. Minimally Invasive Surgery for Lesions Located in Posterosuperior Segments

Three studies compare the results of laparoscopic and open surgery for HCC located in posterosuperior segments [39,40,42]. All of the studies showed a lower morbidity rate and shorter hospital stay in the laparoscopic group. Only Tagaytay et al. found lower blood loss (218.11 vs. 358.92 mL, *p* = 0.046) and shorter operative time (7.03 vs. 11.78 days, *p* = 0.001) in the laparoscopic group. No differences were found in terms of 90-day mortality.

### 3.5. Recurrent HCC

Ten studies were included in the subgroup of repeat LLRs in patients with recurrent HCC with a total of 596 patients, 450 (77.4%) male and 131 (22.6%) female with a median age between 54 (37–66) and 72 (67–79). One hundred and eighty-three patients (95.3%) were Child–Pugh A, and 9/192 (4.7%) were Child–Pugh B. No Child–Pugh C patients were reported in this subgroup (Table 2). Tumor size ranged between 1.25 (0.8–3.5) and 3.8 (3.3–4.5) cm. Only two studies reported on the location of tumors (254 (71.95%) anterior segments and 99 (28.05%) posterior segments) [51,54]. Minor hepatectomies were performed in the vast majority of cases (578, 97.0%) and 248/338 (73.4%) patients underwent a non-anatomic resection. The median time interval from the first operation ranged between 3.9 (0.2–16) and 32 (3–136) months. In 318 (77%) cases, the first operation was performed by open and in 95 (23%), it was performed by laparoscopy. The site of recurrence was described only in two studies and was shown to be ipsilateral in 40 (65.5%) cases and controlateral in 21 (34.5%) cases [49,50]. Operative time ranged between 84 (40–130) and 315 (181–395) min (Table 3). Pringle maneuver was applied in 13/65 (0.2%) cases, which was probably due to difficult surgical anatomy because of re-operation. Blood loss ranged between 10 (10–50) and 283 ± 823 mL, and 41/520 (7.9%) patients required intraoperative blood transfusions. Conversion to laparotomy happened in 22/481 (4.6%). Regarding postoperative outcomes, 109/565 (19.3%) patients developed complications of which 58 (53.3%) were minor and 51 (46.7%) were major (Table 4). Two patients (0.5%) experienced liver failure, 10 (2.3%) developed ascites, and 17 (3.9%) developed a bile leak. The hospital stay ranged between 4 (3–5) and 11.7 ± 11.5 days. Two patients died within 90 days from surgery with a mortality rate of 0.34%.

#### Comparative Results between Open vs. Minimally Invasive Surgery for Recurrent HCC

Eight studies compared the results of laparoscopic vs. open surgery for recurrent HCCs [45,47,49,50,51,52,53,54]. All of them showed a shorter hospital stay in the laparoscopic group. The majority found lower blood loss [45,49,50,51,53] and only three studies reported lower postoperative morbidity rate in the laparoscopic group [45,52,54]. Concerning operative time, Morise et al. and Goh et al. reported longer operative time, while Zhang et al. reported shorter operative time in the laparoscopic group [47,50,51]. Gon et al. showed shorter operative time in the laparoscopic group only if the recurrent HCC was located in the controlateral parenhcyma from the previous resection [49]. No statistically significant differences in 90-day mortality was observed.

## 4. Discussion

Despite the recent advances in surgical techniques and the widespread adoption of minimally invasive approaches for liver resections, patients with advanced cirrhosis, portal hypertension, large and recurrent lesions, and tumors located in the posterosuperior segments still represent a challenge even in the most experienced hands. Indeed, perioperative complications in the above-mentioned settings are potentially high, and long-term outcomes are still under investigation [15]. Careful preoperative evaluation and assessment of potential risk factors is key to guide a thorough discussion of potential risks and benefits, thereby selecting patients and minimizing unexpected events.

Patients with advanced cirrhosis and portal hypertension represent one of the most difficult clinical scenarios in the management of HCC [3]. Indeed, these patients may present with impaired performance status, sarcopenia, encephalopathy, ascites, and severe portosystemic shunts. Therapeutic alternatives such as liver transplantation and locoregional options might come into play, but many patients still undergo resection. The decision of whether to operate on patients with such advanced conditions represents a dilemma. Perioperative risks are high, with increased rates of postoperative morbidity, especially liver failure and ascites [17,55,56]. In this setting, minimally invasive approaches could be beneficial to improve postoperative outcomes [7,9,21]. Indeed, the abdominal cavity is respected as compared to a large open incision, avoiding the interruption of portosystemic shunts, manipulation of the liver is reduced, and the abdominal cavity is not exposed to the air, thus avoiding electrolyte imbalances [57]. However, the LLRs in such patients are technically more challenging. Adhesions are well vascularized, there is an increased bleeding during the transection, and the parenchyma is stiff, thus limiting exposure. According to our review, only four papers have been reported describing LLRs on AC, thus limiting the evidence in this setting. Furthermore, most patients with advanced cirrhosis were scored as Child–Pugh B, while only six patients were scored as C. The literature on liver resection in Child–Pugh C patients is limited both in open and laparoscopic surgery because of the questionable postoperative outcomes [15]. In our opinion, therapeutic alternatives should be well discussed in such patients, as no sufficient data are available so far to support resection, especially in laparoscopy. Although minor and non-anatomical resections were more frequent in these subgroups, intraoperative blood loss was high, the Pringle maneuver was frequently applied (40.4% in AC and 60.6% in PH), and conversion rates were high (8.3% in AC and 9.0% in PH), confirming the technical complexity of these procedures. Despite the potential advantages of the minimally invasive approach, according to our review, AC and PH had the highest rates of morbidity, especially postoperative liver failure (up to 6.7% in PH), ascites production (up to 18.6% in AC) and the highest chance of dying after surgery (5.1% mortality in AC). This confirms that the presence of clinically significant portal hypertension and advanced cirrhosis are important prognostic factors for worse postoperative outcomes, especially in terms of liver decompensation surrogates. For this reason, these very high-risk patients, when considered for surgery, should be managed by experienced surgeons in high volume centers and should be well selected to improve the outcomes. 

Large HCCs represent another common surgical dilemma to approach by laparoscopy. These lesions frequently require major hepatectomies and/or anatomic resections. The dissection of the hilar structures, the large parenchymal transection, the major vasculobiliary structures encountered and the extensive mobilizations require specific learning curve, as each of these steps have specific technical challenges [8,58,59]. This is enhanced when dealing with large lesions, since exposure and mobilization are further limited [60]. Notwithstanding, perioperative outcomes were good with no major blood loss or high rates of conversions to open, and only 20% of patients were developing postoperative morbidity, mostly minor in severity. A cutoff of 5 cm was applied by most of the included studies to define large lesions [29,30,31,32,33,34,35]. Together with the dimensions of the tumor that should be further categorized, we also believe that localization of the lesion should be considered in future studies, as perioperative outcomes could be very different between a lesion located close to the hilum or at the periphery. Dimensions and localization would therefore allow for a more precise selection of patients, thereby improving outcomes.

Posterosuperior segments were initially considered as a contraindication to the laparoscopic approach, being defined as the non-laparoscopic segments [61]. Thanks to the widespread adoption of minimally invasive approaches and to the learning curves, nowadays, lesions in the PS segments are frequently approached by laparoscopy, with good short and long-term outcomes for both benign and malignant lesions [62,63]. However, few reports on HCCs in the PS segments exist, as this still represents a challenging indication, especially in cirrhotic patients. According to our review, intraoperative and postoperative outcomes were good, with a morbidity rate as high as 18.6%, thereby disclosing the safety and efficacy of such approach. However, conversion to open was high (15.5%) as was the need for Pringle maneuver (36%), again stressing the technical complexity and thereby confirming the need for advanced technical skills.

Despite the good long-term outcomes of liver resections for HCC, as much as 70% of patients will experience recurrence of their tumor [3,64]. Salvage liver transplantation, for those eligible, represents a valid treatment. However, repeat liver resection could also be used in selected patients, as outcomes are good both in the short and long-term. According to our review, most resections were minor, reflecting the fact that a parenchymal sparing policy is very important in these patients that have already undergone a previous resection. Unnecessary sacrifice of healthy parenchyma should be minimized. We found that repeat resections for recurrent HCCs require long operative time. This is reasonable considering adhesions from previous surgery that can often be vascularized in cirrhotic patients, thereby prolonging the dissection and exposure as well as preparation of the Pringle maneuver. Indeed, the Pringle maneuver was rarely applied (only 0.2% of cases), reflecting the fact that during repeat resections, the pedicle is difficult to sling given previous maneuvers in the area. This makes the liver transection phase potentially riskier, as bleeding cannot be controlled by hilar clamping.

This systematic review has some limitations; first, it is mainly based on retrospective studies, including mostly small and single-center studies. While the evidence is limited for advanced cirrhosis and portal hypertension, more patients have been reported in the setting of large and recurrent lesions and in posterosuperior segments. The wide inclusion period of the studies might also limit the conclusions, since technical evolutions have happened and are still happening in the field of LLRs. Therefore, we need more data to compare minimally invasive surgery and open surgery in the mentioned situations. In this setting, robotics has been increasingly used in the most recent years: from initial skepticism due to the lack of substantial literature to a worldwide adoption of this technique with similar outcomes as compared to laparoscopy [65]. This review was limited to patients operated on by laparoscopy, and conclusions should therefore not be generalized to robotics. Future studies investigating the role of robotic liver resections in challenging scenarios such as the ones depicted in this review are warranted. Long-term outcomes also have been rarely disclosed in these settings [66,67,68]. Further studies should clarify the oncological safety. To our knowledge, this is the first review that includes all the challenging indications for LLRs for HCC. Only Yin et al. explored the role of LLRs in posterosuperior segments, but no pooled evidence exists concerning AC, PH, large lesions, tumors in the PS segments and repeat LLRs [69]. 

## 5. Conclusions

Laparoscopic liver resections for HCC have good short- and long-term outcomes. Advanced cirrhosis and portal hypertension, large and recurrent tumors and lesions located in the posterosuperior segments are challenging clinical scenarios that should be carefully approached by laparoscopy. Safe short-term outcomes can be achieved provided experienced surgeons and high-volume centers. Advanced cirrhosis and portal hypertension are the riskiest scenarios. The selection of patients is key in these settings. 

## Figures and Tables

**Table 1:** Newcastle–Ottawa scale for quality assessment of the included studies.

Study Authors	Selection	Comparability	Outcome	Total
Cipriani et al. [19]	***	**	***	8
Troisi et al. [20]	****	**	***	9
Cai et al. [21]	***	**	***	8
Beard et al. [22]	****	**	***	9
Lim et al. [23]	****	**	***	9
Guo et al. [24]	****	**	**	8
Molina et al. [25]	***	**	***	8
Zheng et al. [26]	***	**	***	8
Casellas et al. [27]	****	**	***	9
Ruzzenente et al. [28]	****	**	***	9
Kwon et al. [29]	***	**	**	7
Chiang et al. [30]	***	**	***	8
Fu et al. [31]	**	**	***	7
Xu et al. [32]	***	**	***	8
Xiang et al. [33]	****	**	***	9
Levi Sandri et al. [34]	****	**	***	9
Ai et al. [35]	****	**	***	9
Casaccia et al. [36]	****	**	**	8
Xiang et al. [37]	****	**	***	9
Lee et al. [38]	***	**	**	7
Tagaytay et al. [39]	***	**	***	8
Kwon et al. [40]	***	**	***	8
Yoon et al. [41]	****	**	**	8
Xiao et al. [42]	****	**	***	9
Cherqui et al. [43]	***	**	**	7
Levi Sandri et al. [44]	****	**	***	9
Liu et al. [45]	***	**	***	8
Belli et al. [46]	**	**	***	7
Goh et al. [47]	***	**	**	7
Levi Sandri et al. [48]	****	**	***	9
Gon et al. [49]	***	**	***	8
Zhang et al. [50]	***	**	**	7
Morise et al. [51]	***	**	**	7
Kanazawa et al. [52]	***	**	*	6
Onoe et al. [53]	***	**	***	8
Miyama et al. [54]	***	**	***	8

Each * counts as 1 point.

**Table 2:** Baseline characteristics of the included studies.

First Author	Subgroup	Country	Type of Study	No. of Patients	Age	Gender M/F	Child–Pugh A/B/C	MELD Score
Cipriani et al. [19]	Advanced cirrhosis	Italy	Retro	25	66 (23–88)	14/11	0/25/0	NR
Troisi et al. [20]	Advanced cirrhosis	Italy	Retro	100	68 (27–84)	75/25	0/100/0	9 (4–22)
Cai et al. [21]	Advanced cirrhosis	China	Retro	5	60 (27–79)	5/0	0/5/0	NR
Beard et al. [22]	Advanced cirrhosis	USA	Retro	26	60.5 (49–77)	22/4	0/20/6	NR
Lim et al. [23]	Portal hypertension	France	Prosp	18	64 (52–83)	11/7	18/0/0	8 (6–11)
Guo et al. [24]	Portal hypertension	China	Retro	16	50 (29–70)	9/7	12/4/0	NR
Molina et al. [25]	Portal hypertension	Spain	Retro	16	64 (50–75)	11/5	16/0/0	NR
Zheng et al. [26]	Portal hypertension	China	Retro	24	58.5 (54–68)	21/3	18/6/0	NR
Casellas et al. [27]	Portal hypertension	Spain	Retro	31	64 ± 8 *	20/11	31/0/0	NR
Ruzzenente et al. [28]	Portal hypertension	Italy	Retro	89	NR	67/22	67/19/3	NR
Kwon et al. [29]	Large HCC	Republic of Korea	Retro	20	56.1 ± 12.6 *	16/4	NR	NR
Chiang et al. [30]	Large HCC	Taiwan	Retro	37	58 ± 11.7 *	30/7	36/1/0	NR
Fu et al. [31]	Large HCC	China	Retro	14	61.5 (28–77)	10/4	NR	6 (6–7)
Xu et al. [32]	Large HCC	China	Retro	102	52.5 (25–80)	80/22	NR	NR
Xiang et al. [33]	Large HCC	China	Prosp	128	51 ± 11.9 *	109/19	108/20/0	NR
Levi Sandri et al. [34]	Large HCC	Italy	Retro	38	71 (61–77)	25/13	38/0/0	7 (6–8)
Ai et al. [35]	Large HCC	China	Retro	97	52 (14–77)	75/22	59/38/0	NR
Casaccia et al. [36]	Posterosuperior segments	Italy	Retro	22	66 (47–76)	13/9	19/3/0	NR
Xiang et al. [37]	Posterosuperior segments	China	Retro	56	51.6 ± 10.2	47/9	NR	NR
Lee et al. [38]	Posterosuperior segments	Republic of Korea	Retro	58	56 (33–74)	37/21	56/2/0	NR
Tagaytay et al. [39]	Posterosuperior segments	Republic of Korea	Retro	37	60 ± 10.58 *	28/9	NR	NR
Kwon et al. [40]	Posterosuperior segments	Republic of Korea	Retro	149	57 ± 10.4 *	115/34	146/1/2	NR
Yoon et al. [41]	Posterosuperior segments	Republic of Korea	Retro	25	53 ± 10 *	14/11	23/2/0	NR
Xiao et al. [42]	Posterosuperior segments	China	Retro	41	52 ± 11.62 *	34/7	39/2/0	NR
Cherqui et al. [43]	Posterosuperior segments	France	Retro	27	63 (40–76)	22/5	27/0/0	NR
Levi Sandri et al. [44]	Posterosuperior segments	Italy	Retro	62	71 (59.5–75)	50/12	62/0/0	7 (6–8)
Liu et al. [45]	Recurrent HCC	China	Retro	30	56.5 (27–79)	23/7	30/0/0	NR
Belli et al. [46]	Recurrent HCC	Italy	Retro	15	68 (58–75)	NR	15/0/0	NR
Goh et al. [47]	Recurrent HCC	Singapore	Retro	20	68.5 (67–71)	18/2	NR	NR
Levi Sandri et al. [48]	Recurrent HCC	Italy	Retro	74	72 (65–76)	55/19	66/8/0	7 (7–9)
Gon et al. [49]	Recurrent HCC	Japan	Retro	23	72 (67–79)	18/5	23/0/0	NR
Zhang et al. [50]	Recurrent HCC	China	Prosp	31	54 (37–66)	26/5	NR	NR
Morise et al. [51]	Recurrent HCC	Japan	Retro	238	67 ± 11.8 *	181/57	NR	NR
Kanazawa et al. [52]	Recurrent HCC	Japan	Retro	20	70 (46–83)	15/5	19/1/0	NR
Onoe et al. [53]	Recurrent HCC	Japan	Retro	30	71(50–85)	23/7	30/0/0	5 (4–13)
Miyama et al. [54]	Recurrent HCC	Japan	Retro	115	68 ± 10.8 *	91/24	NR	NR

Data are expressed as median (min; max). NR, not reported. HCC, hepatocellular carcinoma. Retro, retrospective. Prosp, prospective. * Data expressed as mean ± standard deviation.

**Table 3:** Intraoperative characteristics of the included studies.

First Author	Subgroup	Type of Hepatectomy Major/Minor	Type of Resection Non-Anatomic/Anatomic	Operative Time (min)	Pringle *n* (%)	Conversion *n* (%)	Blood Loss (mL)	Intraoperative Transfusions *n* (%)
Cipriani et al. [19]	Advanced cirrhosis	NR	NR	210 (120–280)	7 (28%)	4 (16%)	350 (200–1000)	3 (12%)
Troisi et al. [20]	Advanced cirrhosis	14/86	51/49	235 (84–605)	56 (56%)	6 (6%)	110 (0–3270)	1 (1-4)
Cai et al. [21]	Advanced cirrhosis	0/5	5/0	135 (80–170)	0	2 (40%)	800 (240–1000)	NR
Beard et al. [22]	Advanced cirrhosis	0/26	18/8	99 (43–354)	0	1 (4%)	50 (10–4750)	4 (15%)
Lim et al. [23]	Portal hypertension	2/16	12/6	240 (100–360)	NR	2 (11%)	300 (20–1700)	0 (0%)
Guo et al. [24]	Portal hypertension	0/16	16/0	336 ± 18 *	NR	NR	337 ± 351 *	NR
Molina et al. [25]	Portal hypertension	0/15	4/12	150 (90–215)	6 (40)	3 (20%)	90 (80–1000)	1 (7%)
Zheng et al. [26]	Portal hypertension	12/12	15/9	180 (150–250)	12 (50%)	2 (8.3%)	200 (100–400)	5 (21%)
Casellas et al. [27]	Portal hypertension	1/30	17/14	280 (202–338)	25 (81%)	1 (3%)	415 (200–731)	1 (3%)
Ruzzenente et al. [28]	Portal hypertension	14/75	NR	NR	NR	NR	NR	NR
Kwon et al. [29]	Large HCC	11/9	1/19	358.8 ± 136 *	0 (0%)	2 (10%)	600 (NR)	5 (25%)
Chiang et al. [30]	Large HCC	19/18	4/33	232 ± 91.2 *	NR	1 (2.7%)	623 ± 841.75 *	NR
Fu et al. [31]	Large HCC	0/14	0/14	195 (90–390)	NR	1 (7%)	50 (10–1200)	13 (93%)
Xu et al. [32]	Large HCC	28/74	51/51	217.5 (55–470)	50 (0–115) **	3 (3%)	175 (10–1000)	3 (3%)
Xiang et al. [33]	Large HCC	28/100	70/58	234 (105–501)	NR	12 (9.4%)	456 (50–2000)	23 (18%)
Levi Sandri et al. [34]	Large HCC	12/26	9/29	225 (159–270)	10 (26%)	7 (18.4%)	300 (75–800)	NR
Ai et al. [35]	Large HCC	5/92	24/73	245 ± 105 *	NR	9 (9%)	460 ± 426 *	5 (4.5%)
Casaccia et al. [36]	Posterosuperior segments	0/22	15/7	300 (120–560)	1 (4.5%)	1 (4.5%)	55 (20–1400)	10 (45.4%)
Xiang et al. [37]	Posterosuperior segments	14/42	31/25	217.5 ± 63.7 *	NR	10 (17.9%)	295 ± 187 *	9 (16.1%)
Lee et al. [38]	Posterosuperior segments	8/50	16/42	355 (165–930)	NR	8 (13.8%)	600 (130–14,300)	NR
Tagaytay et al. [39]	Posterosuperior segments	0/37	25/12	215 ± 70 *	NR	1 (2.7%)	201 ± 254 *	1 (1.8%)
Kwon et al. [40]	Posterosuperior segments	28/121	73/76	362 ± 180.7 *	60 (40%)	28 (19%)	1376 ± 2509 *	22 (15%)
Yoon et al. [41]	Posterosuperior segments	6/19	7/18	347 ± 117.9 *	NR	4 (16%)	986 ± 920.8 *	10 (40%)
Xiao et al. [42]	Posterosuperior segments	6/35	7/34	242 ± 73.6 *	NR	3 (7.3%)	272 ± 170 *	3 (7.3%)
Cherqui et al. [43]	Posterosuperior segments	1/26	10/17	240 (150–360)	NR	7 (26%)	338 ± 182 *	3 (15%)
Levi Sandri et al. [44]	Posterosuperior segments	12/50	32/30	240 (172–300)	32 (18–45) **	12 (18%)	200 (50–300)	5 (8%)
Liu et al. [45]	Recurrent HCC	1/29	19/11	200.5 (68–525)	0	4 (13.3%)	100 (10–600)	0 (0%)
Belli et al. [46]	Recurrent HCC	0/15	7/8	84 (40–130)	9	1 (6.6%)	NR	NR
Goh et al. [47]	Recurrent HCC	2/18	0/20	315 (181–395)	4 (20%)	3 (15%)	200 (100–450)	2 (10%)
Levi Sandri et al. [48]	Recurrent HCC	5/69	47/27	210 (150–300)	NR	9 (12.1%)	100 (50–225)	5 (6.7%)
Gon et al. [49]	Recurrent HCC	0/23	21/2	286 (251–417)	NR	1 (4%)	10 (10–50)	0 (0%)
Zhang et al. [50]	Recurrent HCC	0/31	19/12	116 ± 37.5 *	NR	0 (0%)	117.5 ± 35.5 *	NR
Morise et al. [51]	Recurrent HCC	9/229	NR	272 ± 187 *	NR	0 (0%)	268 ± 730 *	22 (9%)
Kanazawa et al. [52]	Recurrent HCC	0/20	NR	239 (69–658)	NR	2 (10%)	78 (1–1500)	0 (0%)
Onoe et al. [53]	Recurrent HCC	0/30	27/3	276 (125–589)	NR	2 (6.75%)	100 (0–1050)	NR
Miyama et al. [54]	Recurrent HCC	1/114	108/7	260 ± 158 *	NR	NR	283 ± 823 *	12 (10%)

Data are expressed as median (min–max). NR, not reported. HCC, hepatocellular carcinoma. * Data expressed as mean ± standard deviation. ** Mean time of clamping.

**Table 4:** Post operative outcomes of the included studies.

First Author	Subgroup	Morbidity *n* (%)	CD 0-II *n* (%)	CD III-IV *n* (%)	Liver Failure *n* (%)	Ascites *n* (%)	Bile Leak *n* (%)	Mortality *n* (%)
Cipriani et al. [19]	Advanced cirrhosis	9 (36%)	7 (78%)	2 (22%)	1 (4%)	3 (12%)	1 (4%)	4 (16%)
Troisi et al. [20]	Advanced cirrhosis	38 (38%)	31 (81.5%)	7 (18.5%)	2 (5%)	26 (68.4%)	1 (3%)	2 (2%)
Cai et al. [21]	Advanced cirrhosis	1 (20%)	1 (100%)	0 (0%)	0 (0%)	0 (0%)	0 (0%)	0 (0%)
Beard et al. [22]	Advanced cirrhosis	6 (23%)	5 (19%)	1 (4%)	0 (0%)	0 (0%)	0 (0%)	2 (8%)
Lim et al. [23]	Portal hypertension	7 (39%)	7 (100%)	0 (0%)	2 (28.5%)	2 (28.5%)	0 (0%)	0 (0%)
Guo et al. [24]	Portal hypertension	6 (37.5%)	NR	NR	NR	NR	NR	0 (0%)
Molina et al. [25]	Portal hypertension	6 (40%)	4 (67%)	2 (33%)	0 (0%)	1 (17%)	0 (0%)	0 (0%)
Zheng et al. [26]	Portal hypertension	8 (33%)	5 (62.5%)	3 (37.5%)	1 (12.5%)	2 (25%)	0 (0%)	0 (0%)
Casellas et al. [27]	Portal hypertension	16 (52%)	14 (93%)	2 (7%)	3 (19%)	5 (31%)	0 (0%)	0 (0%)
Ruzzenente et al. [28]	Portal hypertension	26 (29%)	15 (57%)	11 (42%)	NR	NR	NR	0 (0%)
Kwon et al. [29]	Large HCC	3 (15%)	3 (100%)	0 (0%)	0 (0%)	0 (0%)	0 (0%)	0 (0%)
Chiang et al. [30]	Large HCC	7 (18.9%)	6 (85%)	1 (15%)	1 (14%)	3 (43%)	1 (14%)	0 (0%)
Fu et al. [31]	Large HCC	1 (7%)	1 (100%)	0 (0%)	0 (0%)	0 (0%)	0 (0%)	0 (0%)
Xu et al. [32]	Large HCC	20 (19%)	15 (75%)	5 (25%)	4 (20%)	5 (25%)	4 (20%)	0 (0%)
Xiang et al. [33]	Large HCC	26 (20.3%)	13 (50%)	13 (50%)	2 (7.7%)	0 (0%)	0 (0%)	1 (0.78%)
Levi Sandri et al. [34]	Large HCC	20 (52%)	17 (85%)	3 (15%)	0 (0%)	NR	NR	0 (0%)
Ai et al. [35]	Large HCC	10 (10%)	10 (100%)	0 (0%)	0 (0%)	0 (0%)	0 (0%)	0 (0%)
Casaccia et al. [36]	Posterosuperior segments	4 (18%)	4 (100%)	0 (0%)	NR	NR	NR	0 (0%)
Xiang et al. [37]	Posterosuperior segments	9 (16.1%)	9 (100%)	0 (0%)	0 (0%)	1 (11%)	1 (11%)	0 (0%)
Lee et al. [38]	Posterosuperior segments	10 (17.2%)	4 (40%)	6 (60%)	NR	NR	NR	0 (0%)
Tagaytay et al. [39]	Posterosuperior segments	3 (8.1%)	2 (67%)	1 (33%)	0 (0%)	0 (0%)	0 (0%)	0 (0%)
Kwon et al. [40]	Posterosuperior segments	28 (19%)	14 (50%)	14 (50%)	NR	NR	NR	0 (0%)
Yoon et al. [41]	Posterosuperior segments	7 (28%)	7 (100%)	0 (0%)	NR	NR	NR	0 (0%)
Xiao et al. [42]	Posterosuperior segments	7 (17%)	5 (71%)	2 (29%)	0 (0%)	1 (14%)	1 (14%)	0 (0%)
Cherqui et al. [43]	Posterosuperior segments	9 (33%)	NR	NR	1 (4%)	2 (7%)	0 (0%)	0 (0%)
Levi Sandri et al. [44]	Posterosuperior segments	NR	NR	NR	NR	NR	NR	0 (0%)
Liu et al. [45]	Recurrent HCC	2 (6.7%)	1 (50%)	1 (50%)	0 (0%)	0 (0%)	1 (50%)	0 (0%)
Belli et al. [46]	Recurrent HCC	4 (26.6%)	4 (100%)	0 (0%)	0 (0%)	1 (25%)	0 (0%)	0 (0%)
Goh et al. [47]	Recurrent HCC	2 (10%)	2 (100%)	0 (0%)	NR	NR	NR	0 (0%)
Levi Sandri et al. [48]	Recurrent HCC	17 (22.9%)	5 (29%)	12 (71%)	NR	3 (3.7%)	1 (1.7%)	0 (0%)
Gon et al. [49]	Recurrent HCC	2 (9%)	1 (50%)	1 (50%)	0 (0%)	0 (0%)	0 (0%)	0 (0%)
Zhang et al. [50]	Recurrent HCC	NR	NR	0 (0%)	NR	NR	NR	1 (3%)
Morise et al. [51]	Recurrent HCC	36 (15%)	7 (19%)	29 (81%)	2 (5.5%)	5 (14%)	15 (42%)	1 (0.4%)
Kanazawa et al. [52]	Recurrent HCC	1 (5%)	0 (0%)	1 (100%)	0 (0%)	1 (100%)	0 (0%)	0 (0%)
Onoe et al. [53]	Recurrent HCC	30 (100%)	28 (93.3%)	2 (6.7%)	0 (0%)	0 (0%)	0 (0%)	0 (0%)
Miyama et al. [54]	Recurrent HCC	15 (13%)	10 (67%)	5 (22%)	NR	NR	NR	0 (0%)

Data are expressed as median (range). NR, not reported. HCC, hepatocellular carcinoma. CD, Clavien–Dindo.

## Data Availability

Not applicable.
